# Digital versus Traditional Workflow for Immediate Loading in Single-Implant Restoration: A Randomized Clinical Trial

**DOI:** 10.3390/biology10121281

**Published:** 2021-12-06

**Authors:** Paolo Capparé, Francesco Ferrini, Corrado Ruscica, Giuseppe Pantaleo, Giulia Tetè, Enrico Felice Gherlone

**Affiliations:** 1 Dental School, Vita-Salute San Raffaele University, IRCCS San Raffaele, 20132 Milan, Italy; ferrini.f@gmail.com (F.F.); ruscica-corrado@virgilio.it (C.R.); gherlone.enrico@hsr.it (E.F.G.); 2 Department of Dentistry, IRCCS San Raffaele Hospital and Dental School, Vita-Salute San Raffaele University, 20123 Milan, Italy; tetegiulia92@gmail.com; 3 UniSR-Social.Lab, Faculty of Psychology, Vita-Salute San Raffaele University, 20123 Milan, Italy; pantaleo.giuseppe@hsr.it

**Keywords:** digital dentistry, single implant, dental implant, immediate loading

## Abstract

**Simple Summary:**

Immediate loading is nowadays a diffused technique in implantology. At the same time, digital dentistry is rapidly spreading, especially in what concerns prosthetic rehabilitation. The present study aimed to compare, through a randomized clinical trial design, the analogical vs. the traditional workflow. Single-implant restorations have been run in 50 patients, with a 12-month maximum period of follow-up after implant placement. Data were analyzed through radiological, clinical, and customer satisfaction evaluation. Importantly, and surprisingly enough, no statistically significant differences emerged between the two kinds of workflow. When assessing customer satisfaction, however, patients clearly and significantly preferred the comfort of the digital vs. traditional workflow.

**Abstract:**

The purpose of this randomized controlled trial was to compare the immediate-loading protocol, in single restorations in the esthetic zone, by comparing the digital workflow in a test group (TG) vs. the analogical workflow in a control group (CG). A total of 50 patients were enrolled, requiring single hopeless tooth extraction. Twenty-five patients (TG) were randomly assigned to the immediate-loading protocol using the digital workflow, and twenty-five patients (CG) were assigned to the conventional workflow. Clinical and radiographic parameters were evaluated at the time of implant insertion (baseline) and after 3, 6 and 12 months, respectively. A clinician blind to conditions measured the Pink Esthetic Score (PES), as well as patient satisfaction. At 12-month follow-up, a cumulative survival rate of 100% was reported for all implants. No failures or biological complications were observed. No statistically significant differences were detected in the mean values of marginal bone loss and PES between the TG (0.12 ± 0.66 mm for MBL, 7.75 ± 0.89 for PES) and the CG (0.15 ± 0.54 mm for MBL, 7.50 ± 0.89 for PES). In 11 cases of TG, and 10 cases of CG, a one-year follow-up period showed an increased marginal bone level. No statistically significant differences were found in the mean total PES between test (7.75 ± 0.89) and control (7.5 ± 0.81) conditions. Furthermore, a customer satisfaction survey showed that patients preferred the digital workflow over the conventional workflow procedure (97.6 ± 4.3 vs. 69.2 ± 13.8). Digital workflow was more time-efficient than conventional workflow (97.2 ± 7.3 vs. 81.2 ± 11.3). Within the limitations of this study, no statistically significant differences were found between digital and traditional workflow.

## 1. Introduction

Since the advent of modern implantology, surgical and prosthetic protocols have improved over time, leading to predictable treatment outcomes with well-documented long-term implant and prosthetic survival rates [1,2].

Nowadays, implants are medical devices used not only in dental medicine but in many medical fields, such as cardiac implants [3], orthopedic implants [4] and brain implants [5].

Furthermore, implant-prosthetic rehabilitations can be the optimal therapeutic solution in patients with systemic or immunocompromised diseases [6,7].

Some studies [8,9] have shown that placement of implants into the previously grafted sockets is comparable to that into the non-grafted sites.

Various authors [10,11] have described a high rate of success in immediate-loading implant restorations.

In 2004, Pjetursson et al. [12] completed a systematic review that evaluated the survival rate of implant-supported fixed single and partial prosthesis in the esthetic zone.

At five years, the observed survival rate was first 95.4% and then at 92.8% [13]. In a further systematic review of 32 studies run in 2012, the same authors reported a survival rate of 95.6% and 93.1% at 5- and 10-year follow-up, respectively.

In general, the immediate loading protocol makes it possible to receive the fixed dentures within 48 h of implant positioning [13].

This procedure has been highly appreciated by patients, since it avoids the second phase of surgery, decreases treatment time frames and offers immediate comfort for patients who do not need a temporary removable prosthesis during the recovery phase [14].

The traditional analogical workflow for implant-prosthetic restoration has long been selected as the optimal technique in clinical practice.

The accuracy of the impression, which, according to a further review of the literature [15], is the primary factor that influences adaptation to the prosthesis, is typically dependent on the material, the impression technique, the angling of the implant and the number of implants.

The accuracy of the intraoral scanner has been demonstrated by various authors [16,17,18]. This modern technology has gradually attracted increased interest in dentistry [19].

IOS (intraoral scanners) have undergone continuous improvements, and indeed, a study by Bilir et al. [20] confirmed the enhanced effectiveness of digital vs. analogical impressions, not only because digital procedures take less time but also due to the decreased risk of impression material distortion, the 3D preview, and the improved acceptance of that kind of workflow by patients.

The use of the digital method decreases session time since prostheses can be fabricated more rapidly than with the conventional impression technique [21], thereby increasing patient’s acceptance of the impression procedure [22].

Another advantage of the digital impression is the possibility of following an entirely digital workflow thanks to direct interaction with CAD/CAM systems [23,24,25].

Once the intraoral scan has been performed, the CAD-CAM procedures allow to design and produce by milling single prosthetic abutments and temporary and permanent prostheses [26].

However, digital procedures also present some disadvantages [20,21], as for instance the necessity of establishing a learning curve before obtaining satisfactory results. A further disadvantage is represented by the high cost of the devices.

The new available technologies, such as the intraoral scanners and the CAD-CAM technologies, together with the availability of prosthetic materials render the immediate-loading protocol [21] possible, with a remarkable decrease in procedure times, improved patient satisfaction and suitable esthetic results [27,28].

The purpose of this clinical study was to compare the immediate loading protocol, in single post-extractive implants in the esthetic zone, using the digital workflow in a test group (TG) vs. the analogical workflow in a control group (CG). This has been done considering that digital procedures are commonly used in dentistry, but that as yet, the literature includes only a few studies on this specific topic.

## 2. Materials and Methods

This study was designed as a prospective, randomized controlled trial, with a parallel arm design to compare digital versus analogical workflows in an immediate loading protocol, in single post-extractive implants in the esthetic zone.

The study protocol was approved by the Vita-Salute University Institutional Review Committee, Milan, Italy, for human subjects (EC n. 9/INT/2015).

### 2.1. Patients Selection

This clinical study recruited and evaluated patients treated at the Department of Dentistry, IRCCS San Raffaele Hospital, Milan, Italy, from March 2017 to May 2018. 

The target population was represented by men and women over 18 years old, requiring single tooth extraction in the anterior maxillary or mandibular areas (esthetic area, from the second premolar to the second premolar) due to tooth failure, periodontal disease, root fracture or destructive decay.

Inclusion and exclusion criteria are reported in Table 1.

The protocol for the RCT was explained in detail to all patients, who subsequently first read and then signed an informed consent form. 

This was obtained before the inclusion screening process.

### 2.2. Sample Size and Randomization of Participants

Prior to data collection, a sample size of 50 patients was considered acceptable to reach the required level of statistical power. 

To be able to detect an effect size (ES) of at least f = 0.50 in a mixed 2 × 4 MANOVA, a total sample size of N = 48 (then rounded to 50) was required (statistical power = 80%; alpha = 0.05), this leading to *n* = 25 participants per study group. 

**Figure 1:**
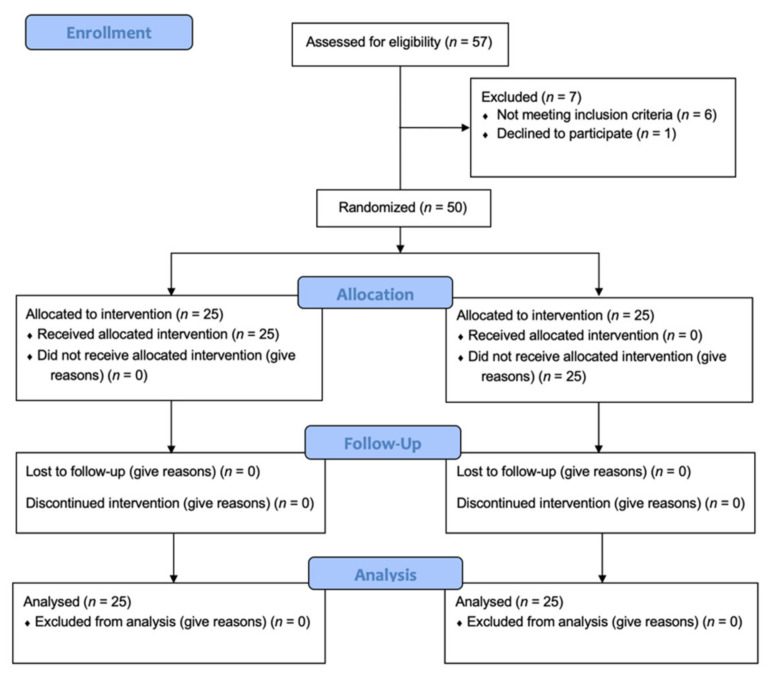
Flowchart of the controlled clinical trial protocol used in this study based on the Consolidated Standards of Reporting Trials (CONSORT 2010).

A total of 57 patients were initially screened for participation in the study, but only 50 were included (Figure 1).

Twenty-five patients were randomly assigned to the digital group (TG), and 25 patients to the analogical group (CG).

A collaborator not involved in the study prepared a computer-generated randomization list. 

The randomization code was closed in an opaque envelope numbered sequentially, then opened by a research assistant, after the implant was inserted and the required torque achieved (35 N/cm) (Figure 1).

### 2.3. Preoperative Evaluation

During the first dental visit, periodontal screening and recording (PSR) was performed to assess patients’ oral hygiene and periodontal health.

Patients with a PSR score >3 were treated with non-surgical etiological therapy and reconsidered after 1 month. Patients with a plaque index (PI) over 2 were excluded from the study.

Each clinical case was examined carefully, by evaluating diagnostic models, preoperative panoramic and intraoral radiograph, and cone beam computed tomography.

### 2.4. Pretreatment

All patients took two tablets of Amoxicillin 875 mg + Clavulanic acid 125 mg (Augmentin, Glaxo Smith Kline, Brentford, UK) 1 h before the procedure.

Before starting with the procedure, patients rinsed their mouth with chlorhexidine at 0.2% [29,30] (Corsodyl, GlaxoSmithKline, Rixensart, Belgium) for one minute. 

### 2.5. Post-Extraction Surgical Protocol

All surgical procedures were performed by a single operator (PC).

**Figure 2:**
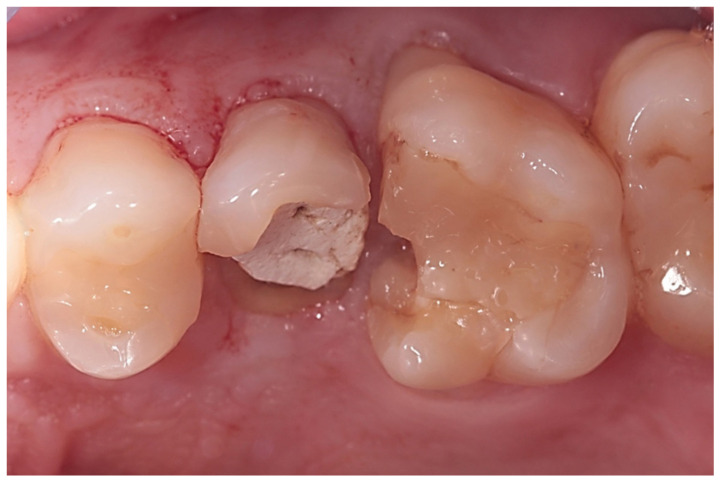
Clinical view of hopeless premolar before extraction.

After local anesthesia with articaine hydrochloride 1:100,000 (Ubistesin 40 mg/mL, 3M ESPE, Milan, Italy), the hopeless teeth were extracted a-traumatically, maintaining the integrity of the fresh socket and, specifically, the buccal bone wall (Figure 2).

A periodontal probe (Hu-Friedy PGF-GFS, Hu-Friedy, Chicago, IL, USA) was used to confirm the integrity of the four socket walls. All experimental sites displayed no fenestration or dehiscence, and no regenerative procedures were completed at any of the sites.

The implant sites were prepared according to the manufacturer’s (CSR implant System, Sweden & Martina, Due Carrare, Padua, Italy) surgical protocol, with standard drills following the palatal wall as a guide, and the apical portion of the implant was prepared 4 mm beyond the apex of the root.

The coronal margin of the implant was located at 0.5 mm apically to the buccal level of the bone crest.

The quality of alveolar bone was evaluated during surgery for each site.

If the gap between implant surfaces and socket walls exceeded 2 mm, small autogenous bone chips were inserted in that space.

### 2.6. Provisional Restoration

To achieve adequate primary stability with an immediate-loading protocol, all implants were inserted with a minimum insertion torque of 35 N/cm.

After the surgical procedure, a temporary abutment was inserted and screwed at 32 N/cm using a dynamometric wrench.

Suturing was completed with simple interrupted stitches using 3/0 absorbable sutures (Vicryl, Ethicon, Johnson & Johnson, New Brunswick, NJ, USA).

After implant insertion, 25 patients were assigned randomly to the immediate loading protocol using the digital workflow (TG) and 25 patients to the analogical workflow (CG).

Randomization occurred through a computer-generated randomization list.

Occlusion was checked and, if necessary, the abutment was personalized or cut and modified outside of the oral cavity.

With respect to the test group (TG), impressions were recorded using the CAD/CAM chairside system (Cerec Omnicam, Dentsply Sirona, York, PA, USA), a system that enables the processing of images and the design of the prosthesis.

Then, the generated file was sent to the Sirona Cerec MCXL milling machine (MCX5, Dentsply Sirona^®^, York, PA, USA) for fabrication of the temporary prostheses in PMMA (Telio CAD, Ivoclar Vivadent AG, Schaan, Liechtenstein).

With respect to the control group (CG), an analogical flow procedure was implemented, whereby temporary prefabricated acrylic resin crowns were obtained and then adapted with an auto-polymerizing acrylic resin (Duralay pattern resin, Reliance Dental Mfg. Co., Worth, IL, USA) along the margins of the temporary abutment.

In both groups, temporary prosthetics were delivered within four hours of the surgical procedure.

Correct static and dynamic occlusion parameters were also assessed, using articulating papers (Bausch Articulating Paper). 

### 2.7. Post-Surgical Instruction

For the entire duration of the post-operative period, all patients were prescribed:

- 1 g of Amoxicillin 875 mg + Clavulanic acid 125 mg (Augmentin, Glaxo Smith Kline, Brentford, UK) twice a day for a period of five days after the procedure;
- 0.20% chlorhexidine mouthwash (Corsodyl, GlaxoSmithKline, Rixensart, Belgium) rinse two times a day for around one minute for the next 15 days;
- Soft diet for the two months after the surgical;
- The light smokers were remembered to limit and possibly to refrain from smoking.

### 2.8. Definitive Restoration

Four months after implant placement, temporary crowns and abutments were removed. 

In the TG group of patients, final impressions were recorded using an intraoral scanner (Cerec Omnicam, Dentsply Sirona, York, PA, USA) after scanbody positioning.

These digital impressions, in the form of STL files, were sent to the laboratory for the realization of the final prosthesis.

In the CG group of patients, transfer copings were positioned and mono-component mono-phase impressions made with a polyether (Impregum Penta, 3M ESPE, Milan, Italy) were taken using the pick-up technique.

The antagonist arch impressions were taken in irreversible hydrocolloid (Alginate CA37 Fast Set, Cavex Holland BV, Harleem, The Netherlands).

**Figure 3:**
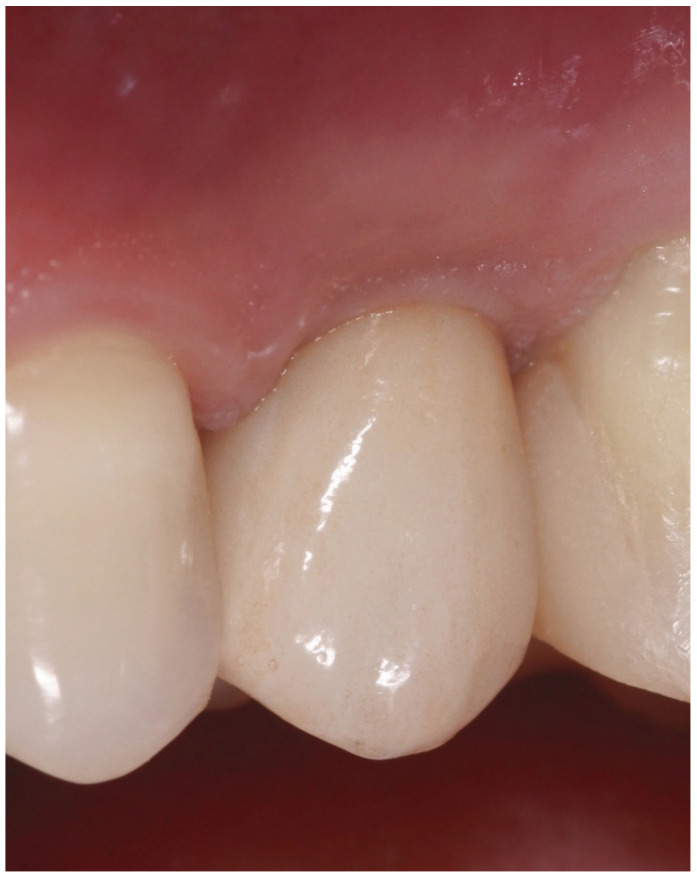
Clinical view of definitive restoration at 12-month follow-up.

Then, the impressions were sent to the laboratory for the realization of the final prosthesis in zirconia ceramic (Figure 3).

### 2.9. Follow-Up and Measurement of Periodontal Clinical Parameters

A single operator, ‘blind’ to experimental conditions, performed all measurements.

All patients were evaluated at the time of the procedure (i.e., at baseline) and then at 3 (T1), 6 (T2) and 12 months (T3), respectively.

Intraoral X-rays were completed as described below.

The measurement of periodontal parameters was performed with a probe (Florida perio-probe, Florida probe Corporation, Gainesville, FL, USA) while also measuring the applied pressure, that should be 0.35 N in all cases.

The following clinical parameters were recorded [31]:

- Height of the keratinized mucosa (KM): measured at the exact middle of the vestibular face of the tooth, using a probe, from the mucogingival junction to the coronal margin of the free gingiva;
- Modified plaque index: measured according to the following scale 0 = no plaque; 1 = presence of plaque; four surfaces (distal, mesial, buccal and lingual) were considered, and the average value for each implant was thus calculated.
- Modified bleeding index (mBI): measured by assessing the amount of bleeding by gentle probing on four surfaces (distal, mesial, buccal, and lingual), according to the scale 0 = no bleeding; 1 = presence of bleeding; the average value for each implant was considered;
- Probing depth (PD): the deepest site measured on the four surfaces of the implant (buccal, lingual, mesial, distal) was considered and approximated to the millimeter.

Systematic variations in the above factors were compared to the baseline levels, and also variations between the two groups were evaluated in a mixed 2 × 4 MANOVA design (see statistical methods below).

### 2.10. X-ray Evaluation

Digital intraoral X-rays (DIGORA, Soredex, Tuusula, Finland) were completed before and after the extraction, at the time on of implant placement (baseline), and at each follow-up visit (3 months, 6 months and 12 months after implant loading).

Periapical X-rays were taken with the parallel long-cone technique, using a standardized film holder (Rinn center XCP Evolution 2003, Dentsply, Italy); a customized occlusal bite jig in acrylic resin was fabricated for obtaining standardized intra-oral periapical radiographs and in order to measure the levels of crestal bone.

An operator ‘blind’ to experimental conditions measured marginal bone levels variation over time, twice a day, on two different days.

The mean of the two measurements, rounded to two decimal places, was used as a reference value.

The reference points and lines measured were reported on the interactive monitor using a specific software (DIGORA, Soredex, Tuusula, Finland).

The length of the implant was used to calibrate the measurement software.

The linear distance between the most coronal point of bone-to-implant contact and the coronal margin of the implant collar was measured to the nearest 0.01 mm, at both mesial and distal sides, and then averaged.

The difference between the various levels of crestal bone in the mesial and distal sides were measured over time using the same software.

All radiographies were evaluated by two independent raters. The intra- and extra- examiner errors were calculated by comparing the first and second measurements with a paired *t*-test at a significance level of 5%. In each case, no statistically significant differences emerged between pairs of values (*p*_s_ > 0.05).

### 2.11. Esthetic Evaluation and Patient Satisfaction

Twelve months after the surgery, the esthetic result was evaluated by an evaluator ‘blind’ to treatment, on the basis of the Pink Esthetic Score (PES) modified by Belser in 2009 [32].

The following PES parameters were then evaluated:

A. The presence/absence of interproximal papilla;
B. The scallop of the gingival margin;
C. The position of the marginal tissue;
D. The color of the marginal tissue;
E. The appearance of the peri-implant tissue.

A score was assigned to each parameter, ranging from 0 (minimum value) to 2 (maximum value).

A score of less than 6 corresponded to unacceptable esthetics, while a score of 6 to 8 represented an esthetically pleasing result; a PES score of 9 or more indicated an excellent esthetics.

Patient satisfaction was evaluated using a questionnaire and a visual analogue scale (VAS) ranging from 0–100, after a time lapse of 12 months [33]. Ten questions were included in the questionnaire as shown in Table 2.

### 2.12. Measurement of Key Clinical Study Variables

- Prosthetic failure: prosthesis not positioned due to implant failure.
- Implant failure: any implant removed because of lack of stability, infection or fractured beyond repair.
- Complications or secondary effects of any type, biological and/or prosthetic
- Peri-implant marginal bone level variations measured mesially and distally using the method described in the section “X-ray evaluation”.
- Periodontal clinical parameters: height of the keratinized mucosa (KM), modified Bleeding Index (mBI), modified Plaque Index (mPI), Probing Depth (PD).

All measurements were performed at the time of implant insertion and 3 months, 6 months and 12 months after loading.

### 2.13. Statistics

The collected data were examined according to a predetermined plan of analysis.

The statistical unit was the patient.

A dedicated software (SPSS 25.0, SPSS Inc., Chicago, IL, USA) was used for statistical analyses. 

The insertion torque value was recorded for the two groups at the time of implant insertion.

An evaluation of radiographic bone levels was also completed. The radiographic bone level measurements (mesial, distal, and mean bone loss) were calculated for each implant and reported as mean ± standard deviations at baseline and at 3, 6 and 12 months after implant positioning, respectively.

The intra-examiner error was calculated by comparing the first and second measurements with a paired *t*-test at a significance level of alpha = 5%.

A multivariate analysis of variance (MANOVA) was computed to compare both the changes that occurred over time in the two groups (i.e., the within-subjects factor), as well as to compare the two groups with each other (i.e., the between-subjects factor). 

Finally, patient satisfaction was assessed by analyzing the VAS scores. 

The threshold value for determining statistical significance of the observed results was conventionally set at alpha = 0.05.

## 3. Results

Fifty-seven patients were assessed for eligibility in the present study. Of 57 patients, 7 were excluded (Figure 1).

Fifty patients thus took part in the study: 31 women and 19 men (Table 3).

At the time of implant insertion, patients were between 23 and 65 years of age (mean age: 45.13 ± 12.4 years).

The entire group of patients completed the treatment (i.e., participated until the end of the study).

Only 22 patients were light smokers. During the period of follow-up, all implants were clinically osseointegrated and showed no signs of infection.

The insertion torque values the day of the surgery were 39.60 ± 11.32 N/cm and 36.40 ± 13.19 N/cm, respectively, for the CG and TG groups.

After a follow-up period of 24 months, a success and survival rate of 100% was observed in both groups. No abutment screw loosening, neither prosthetic nor implant failures, were observed.

### 3.1. Clinical Parameter Evaluation

Clinical parameter values are reported in Table 4. 

Tests of between subject effects (Table 5) revealed a significant influence of type of technique on only mBI measurements (*p* = 0.032).

The factor relating to the time period in which the assessment took place, however, only showed a significant effect on KM measurements (*p* < 0.001) and PD measurements (*p* < 0.001).

The interaction of ‘type of technique’ and ‘period of time’ showed a significant effect on KM measurements (*p* = 0.05), and PD measurements (*p* = 0.05).

### 3.2. Radiographic Parameter Evaluation

The results of the radiographic evaluation are reported in Table 6 and Table 7.

The TG and CG groups showed good maintenance of bone levels, as shown by a mean bone loss at the 12-month follow-up of 0.12 ± 0.66 mm for the TG group and 0.15 ± 0.54 mm for the CG group.

No statistically significant differences were found in the radiographic evaluation either as a function of group (digital vs. analogical technique), or as a function of time of measurement (*p_s_* > 0.05).

Eleven cases in the TG group and 10 cases in the CG group showed an increased marginal bone level at the 12-month follow-up (Table 8).

The mean marginal bone increase was 0.52 ± 0.25 mm in the TG group and 0.41 ± 0.25 mm in the CG group.

### 3.3. Pes Evaluation

PES scores for both groups are reported in Table 9. 

In the test group (TG), the presence of mesial and distal papilla scored an average of 1.54 ± 0.51, and 1.25 ± 0.44, respectively; the scallop and the position of the soft tissue margin in relation to the margin of the prosthetic restoration achieved a score of 1.86 ± 0.36 for both parameters; finally, the color of the soft tissue was 1.25 ± 0.52 for an overall total PES of 7.75 ± 0.89.

In the control group (CG), however, the presence of mesial and distal papilla showed mean scores of 1.71 ± 0.46 and 1.39 ± 0.50, respectively; the scallop of the soft tissue margin showed a score of 1.75 ± 0.44; the position of the soft tissue margin in relation to the margin of the prosthetic restoration reached a mean score of 1.57 ± 0.50; finally, the color of the soft tissue was 1.07 ± 0.60, for an overall PES of 7.50 ± 0.89.

An independent samples *t*-test was also run, showing no statistically significant differences between the two groups (digital vs. analogical) with respect to the total PES values, t (54) = 1.06, *p* = 0.30.

### 3.4. Patients Satisfaction

The VAS scores referring to patient satisfaction in each treatment group (digital vs. analogical flow) are reported in Table 10. 

During the acquisition of impressions, the group assigned to the digital procedure reported less discomfort (mean VAS 96.8 ± 6.42 in the TG group and 72.8 ± 16.57 in the CG group, *p* = 0.007), with nearly absent vomiting reflex/nausea (mean VAS 94.76 ± 9.45 in the TG group and 86.34 ± 10.34 in the CG group, *p* = 0.03), with a subsequently reported higher level of general comfort during the impression procedure (mean VAS 97.6 ± 4.3 in the TG group and 69.2 ± 13.8 in the CG group, *p* = 0.005).

Furthermore, the digital protocol received the highest scores in terms of treatment (mean VAS 97.2 ± 7.3 for the TG group and 81.2 ± 11.3 for the CG group, *p* = 0.023).

There were no differences in satisfaction with esthetic and functional results, with both methods totaling scores of over 90. 

Overall, sheer patient satisfaction (i.e., not comfort and discomfort scores) was nearly equivalent between the two groups (mean VAS 94.32 ± 8.61 for the TG group and 92.02 ± 8.87 for the CG group, *p* = 0.363).

Finally, no statistically significant differences were found in overall mean VAS scores (*p* > 0.05).

## 4. Discussion

The immediate replacement of a single tooth with an implant-prosthetic rehabilitation in the esthetic zone is considered, today, one of the most frequent indications of modern implantology, with high percentages of survival and success, as reported in the literature [33].

The scientific literature suggests alveolar ridge preservation (ARP) with graft materials [34] with or without immediate implant placement as surgical procedures to avoid dimensional changes observed after tooth extractions.

The use of modern digital impression techniques appears to represent an ideal application in implant prosthodontics, especially in the immediate loading restorations, as recently demonstrated in several studies [20,21,24,25].

The present study shows that analogic and digital workflows can both lead to positive, comparable clinical results for what concerns the immediate loading procedure. Ad-hoc planned future studies, however, should try to overcome some of the limitations of this randomized trial. For instance, they could focus on a bigger sample size, to be able to detect even smaller, more nuanced clinical differences between the two conditions of digital vs. traditional workflows, if deemed relevant. Besides increasing the total number of enrolled patients, future research could also make a significant effort in monitoring the clinical vs. subjective outcomes of the two procedures in a longer time span—i.e., one encompassing also an early follow-up period. 

With respect to the uniqueness of our findings, as far as we know, the present research represents the only randomized clinical trial on the topic of digital workflow and immediate loading in single-implant restoration. Moreover, this research established an important asymmetry between the clinical comparability of the two procedures (digital vs. traditional workflow) as to relevant practical effects (i.e., effects involving quite important effect sizes), on the one hand, and a clear preference for the digital vs. the traditional workflow in patients’ own perceptions and judgments. One the other hand, an obvious limitation of our choice to enroll just the needed number of patients (i.e., 25 per experimental condition) to secure the needed statistical power to be able to detect quite relevant (i.e., practically meaningful) effects in a mixed 2 × 4 MANOVA research design can be questioned, if interested in detecting even smaller, more nuanced (clinical and/or subjective) results. This limit can be easily fixed by future research by running a set of power analyses intended to detect much smaller effects, if deemed necessary. 

Considering also the significant interest in the subject and the few papers available, this randomized controlled trial (RCT) recorded a success and a high survival rate of 100% in both groups. No failures or biological complications were observed.

A key element of this positive result was the significant stability of peri-implant bone tissue during the study, with a minimal variation in mean bone loss values (MBL) in both groups. These results are consistent with those of other studies in the literature [35,36,37].

No statistically significant differences were observed between the two groups (*p* > 0.05), and no mean bone loss values over 1.5 mm were reported on the follow-ups at 3, 6 and 12 months in both groups.

In addition, in 11 cases of the TG group and 10 cases in the CG group, the follow-up at 12 months showed an elevated stability of the hard tissues, even with increased levels of stability over time.

The mean marginal bone increase was higher in the TG group than in the CG group (0.52 ± 0.25 mm vs 0.41 ± 0.25 mm).

The present data suggest the possibility of minimizing marginal bone loss or even stimulating an increase around implants, thanks to the morphology of this kind of implant. The double conical connection provides elevated mechanical stability [38,39] and seems to be resistant against bacterial microleakage [40]. All of these could prevent the resorption of crestal bone around the implants [40,41,42].

Related to this, it has also been demonstrated that the stability of crestal bone around implants depends largely on the creation of a biological width suitable for the interface between abutment and implant [43]. 

With this double conical connection, the emergence profile of the implant abutment is different and offers all the characteristic advantages described with the modern principle of platform switching. In various studies on animals [44,45] and humans [46], radiographic and histological analyses have confirmed that platform switching minimizes crestal bone resorption, enabling the long-term maintenance of the crestal apex. 

In the present study, the remarkable stability of the bone crest over time was accompanied by excellent soft tissue health, with low levels of inflammation of the peri-implant tissues.

Inflammation must be controlled to ensure the long-term success of implant rehabilitation; this is especially important for patients with chronic systemic viral or autoimmune diseases [35,47,48,49].

As concerns the KM clinical parameters, the analysis of variance (MANOVA) showed that the two groups differed significantly from each other over time (*p* < 0.05).

From a clinical perspective, this is not of great importance, however, since the measurement method involves the use of a periodontal probe calibrated to the millimeter, and thus, such an observed small variation (mean KM 3.03 ± 1.03 mm for the CG group and 2.57 ± 0.83 mm for the TG group) becomes uninfluential.

Therefore, probably for this reason, the clinical and radiographical results of this randomized study demonstrated, after 1-year follow-up, that both the digital and analogical workflows can positively influence the prognosis of immediate-loading implant-prosthetic restorations.

With respect to the results pertaining to more ‘esthetic’ factors in our study, evaluated by an observer who was blind to the treatment provided, we observed a PES value of 7.75 ± 0.89 in the TG group and of 7.5 ± 0.81 in the CG group, which indicates a completely satisfactory although not superlative esthetic. 

This is not surprising, in our opinion, since PES is primarily influenced by local anatomy and the surgical technique, which highlights how the protocol and the surgeon’s skills in implantation play an important role in achieving desired esthetic results on peri-implant soft tissues. 

None of the 56 implants showed values < 6; this fact confirms the high reliability of the implant-prosthetic protocol used in the present study. 

The low PES scores observed for inter-implant papillae should not be surprising because, as clearly demonstrated in previous clinical studies [37,50,51], the height of the inter-implant papillae primarily depends on the bone level height of the adjacent root surfaces.

No statistically significant differences were then observed between the test group and the control group in mean PES values; this result is in line with the idea that the immediate protocol with digital workflow does not seem to compromise the esthetic result of the implant.

Finally, as shown in other studies [22,23], the digital protocol seemed to be more time-efficient than the conventional protocol. 

## 5. Conclusions

The clinical and radiographic results of this randomized clinical trial, with a year of follow-up and fifty implants inserted, revealed no statistically significant differences between the digital vs. analogical workflow protocol followed in immediate-loading single implant-prosthetic restorations.

Patients’ own esthetic evaluation showed, by contrast, satisfactory results that were fully compatible with those reported in the literature. Specifically, the customer satisfaction analysis indicated greater satisfaction, in terms of comfort/discomfort, with the digital protocol.

Still, further long-term prospective clinical studies, e.g., studies with a greater number of systematically monitored implants, are needed to investigate the full potential of the digital procedure, and to corroborate these preliminary findings. 

## Figures and Tables

**Table 1:** Criteria used for inclusion or exclusion in the study.

Inclusion Criteria	Exclusion Criteria
1. Man/woman	1. Patients who were pregnant or breastfeeding
2. Age > 18 years	2. Radiation therapy on the head/neck or chemotherapy in the last five years
3. Good oral hygiene	3. Poor oral hygiene (Full Mouth Plaque Score (FMPS) and Full Mouth Bleeding Score (FMBS) >20%) and low motivation to maintain correct oral hygiene
4. FMPS and FMBS < 20%;	4. Chronic abuse of drugs or alcohol
5. Non-smokers or smokers of less than 10 cigarettes/day	5. Heavy smokers (>10 cigarettes per day)
6. No contraindications to oral surgery (ASA-1/ASA-2)	6. Compromised medical conditions (ASA score 3 or more)
7. Bone volume and density that would allow for the insertion of an implant with a minimal diameter of 3.0 mm and a minimal length of 8.5 mm	7. The need for bone increase/bone regeneration procedures for the positioning of an implant
8. No active infections around the surgical site	8. Limited mouth opening, functional limitations or temporomandibular problems
9. Signing of an informed consent for the protocol	9. Severe parafunctions (bruxism, clenching)
	10. Systemic medical conditions that would contraindicate implant surgery (e.g., uncontrolled diabetes, coagulation problems not adequately treated and psychiatric problems)
	11. Use of oral and/or parenteral bisphosphonates for >3 years
	12. Immunosuppressant therapy

**Table 2:** Patient satisfaction assessment questionnaire.

Questions
1. Are you satisfied with the treatment? with 0 = very dissatisfied and 100 = very satisfied
2. Are you pleased with the functional result? with 0 = not pleased and 100 = very much pleased
3.Are you pleased with the final aesthetic result? with 0 = not pleased and 100 = very much pleased
4. Did you experience discomfort during the impression-taking? with 0 = high discomfort and 100 = no discomfort
5. Did you experience gag reflex/nausea during the impression-taking? with 0 = strong gag reflex/nausea, and 100 = no gag reflex/nausea
6. How annoying was the impression procedure? with 0 = very annoying and 100 = not annoying
7. Was the treatment time justified? with 0 = totally not justified and 100 = completely justified
8. Has the dental implant treatment performed as expected? with 0 = absolutely not and 100 = yes
9. Would you repeat this treatment again, if necessary? with 0 = absolutely not and 100 = yes, of course
10. Would you encourage friends or family members to perform the same treatment? with 0 = absolutely not and 100 = Yes, obviously

**Table 3:** Composition of the selected sample composition.

	Immediate Loading with Digital Workflow	Immediate Loading with Analogical Workflow
**Men**	14	5
**Women**	11	20
**Overall Patients**	25	25
**Mean Age**	49.21 ± 9.07	41.05 ± 15.73
**Inserted Implants**	25	25

**Table 4:** Clinical parameters at the 12-month follow-up (N implants = 50) (TG = Test Group; CG = Control Group).

	TG				CG			
**Parameter**	Baseline	3 Months	6 Months	12 Months	Baseline	3 Months	6 Months	12 Months
**mPI**	1.07 ± 0.90	0.75 ± 0.70	0.89 ± 0.68	1.03 ± 0.64	0.86 ± 0.93	0.46 ± 0.64	0.64 ± 0.68	0.82 ± 0.77
**mBI**	1.14 ± 0.93	0.64 ± 0.68	0.82 ± 0.81	0.96 ± 0.69	0.82 ± 1.09	0.36 ± 0.49	0.57 ± 0.63	0.78 ± 0.74
**PD (mm)**	2.10 ± 0.87	1.97 ± 0.75	2.21 ± 0.98	2.41 ± 0.89	2.23 ± 0.76	1.87 ± 0.73	2.60 ± 0.65	2.95 ± 1.00
**KM (MM)**	3.25 ± 1.32	2.96 ± 1.17	2.68 ± 1.02	2.57 ± 0.83	3.33 ± 0.69	3.07 ± 1.01	2.89 ± 0.99	3.03 ± 1.03

**Table 5:** Results based on tests of subjects effects, significant effects (α = 0.05) of type of technique and period of time on assessed clinical factors (KM, mBI, mPI and PD) are reported in bold.

Source	Dependent Variable	SUM OF Squares	Degrees of Freedom	Mean Square	F	Significance
**Type of Technique**	KM (mm)	2.161	1	2.161	0.55	0.463
	mBI	3.754	1	3.754	4.832	0.032
	mPI	3.254	1	3.254	2.881	0.095
	PD (mm)	3.135	1	3.135	1.92	0.17
**Times**	KM (mm)	6.914	1	6.914	17.52	<0.001
	mBI	0.044	1	0.044	0.046	0.831
	mPI	0.008	1	0.008	0.014	0.906
	PD (mm)	11.401	1	11.401	20.32	<0.001
**Interaction Type of Technique by Time**	KM (mm)	1.575	1	1.575	3.99	0.05
	mBI	0.151	1	0.151	0.158	0.693
	mPI	0.001	1	0.001	0.002	0.969
	PD (mm)	2.100	1	2.100	3.743	0.05

**Table 6:** Mean marginal bone levels measured for TG and CG.

	Test Group	Control Group
**3 months**	0.04 ± 0.51 mm	0.32 ± 0.52 mm
**6 months**	0.24 ± 0.58 mm	0.25 ± 0.59 mm
**12 months**	0.12 ± 0.66 mm	0.15 ± 0.54 mm

**Table 7:** Marginal bone loss at the 12-month follow-up in the control group (CG).

Bone Loss	Mesial		Distal		Mean Bone Loss	
	*n*	%	*n*	%	*n*	%
<0.0	10	35.71%	11	42.86%	10	35.71%
0.0 a 0.1	5	17.86%	4	14.29%	3	10.71%
0.1 a 0.5	6	21.43%	5	17.86%	6	28.57%
0.6 a 1.0	3	14.29%	3	17.86%	5	21.43%
1.1 a 2.0	1	10.71%	2	7.14%	1	3.57%
>2.0	0	0%	0	0%	0	0%
<0.0	12	42.86%	9	35.71%	11	39.28%
0.0 a 0.1	2	7.14%	3	14.29%	2	7.14%
0.1 a 0.5	8	35.71%	8	32.14%	6	28.57%
0.6 a 1.0	0	0%	2	7.14%	4	17.86%
1.1 a 2.0	3	14.29%	3	10.71%	2	7.14%
>2.0	0	0%	0	0%	0	0%

**Table 8:** Values of marginal bone increase in the test group (TG) and in the control group (CG).

Mean Marginal Bone Increase	
Test Group	Control Group
0.24 mm	0.05 mm
0.28 mm	0.21 mm
0.29 mm	0.26 mm
0.32 mm	0.29 mm
0.36 mm	0.30 mm
0.57 mm	0.33 mm
0.58 mm	0.50 mm
0.64 mm	0.64 mm
0.66 mm	0.64 mm
0.70 mm	0.89 mm
1.09 mm	

**Table 9:** PES values for the test group (TG) and control group (CG).

PES Value	Group	Mesial Papilla	Distal Papilla	Scallop of the Soft Tissue Margin	Level of Facial Mucosa	Soft Tissue Color and Texture
**Maximum**	TG	2	2	2	2	2
	CG	2	2	2	2	2
**Minimum**	TG	1	1	1	1	0
	CG	1	1	1	1	0
**Mean ± SD**	TG	1.54 ± 0.51	1.25 ± 0.44	1.86 ± 0.36	1.86 ± 0.36	1.25 ± 0.52
	CG	1.71 ± 0.46	1.39 ± 0.50	1.75 ± 0.44	1.57 ± 0.50	1.07 ± 0.60

**Table 10:** Mean and standard deviation of visual analog scale (VAS) scores tapping into patients’ satisfaction.

Questionnaires	Group	Mean + SD	*t*-Test *p* Value
**Question 1**	TG	94.32 ± 8.61	0.363
	CG	92.02 ± 8.87	
**Question 2**	TG	93.2 ± 8.01	0.59
	CG	92 ± 7.6	
**Question 3**	TG	95.05 ± 8.56	0.161
	CG	91.97 ± 10.32	
**Question 4**	TG	96.8 ± 6.42	0.007
	CG	72.8 ± 16.57	
**Question 5**	TG	94.76 ± 9.45	0.03
	CG	86.34 ± 0.34	
**Question 6**	TG	97.6 ± 4.3	0.005
	CG	69.2 ± 13.8	
**Question 7**	TG	97.2 ± 7.3	0.023
	CG	81.2 ± 11.3	
**Question 8**	TG	94.99 ± 9.05	0.450
	CG	93.11 ± 9.39	
**Question 9**	TG	91.2 ± 8.8	0.73
	CG	90.4 ± 7.3	
**Question 10**	TG	92.8 ± 7.9	0.86
	CG	92.4 ± 8.3	

## Data Availability

The data presented in this study are available on reasonable request from the corresponding author.
